# Highly effective transformation of methyl phenyl carbonate to diphenyl carbonate with recyclable Pb nanocatalyst†

**DOI:** 10.1039/c9ra03931g

**Published:** 2019-07-01

**Authors:** Songlin Wang, Hongying Niu, Jianji Wang, Tong Chen, Gongying Wang, Jiamin Zhang

**Affiliations:** School of Chemistry and Chemical Engineering, Henan Institute of Science and Technology Xinxiang 453003 P. R. China wangsonglin2009@163.com; Postdoctoral Programs, Key Laboratory of Green Chemical Media and Reactions, Ministry of Education, School of Chemistry and Chemical Engineering, Henan Normal University Xinxiang 453007 P. R. China jwang@htu.cn; Chengdu Institute of Organic Chemistry, Chinese Academy of Sciences Chengdu 610041 P. R. China chentongw@sina.com.cn

## Abstract

Diphenyl carbonate (DPC) is a type of versatile industrial chemical, and the disproportionation of methyl phenyl carbonate (MPC) is a key step to produce DPC. However, the design and formulation of a catalyst for the efficient synthesis of DPC is a major challenge due to its small equilibrium constant. The support material is a critical factor influencing the performance of Pb nanocatalysts. Thus, a series of Pb-based catalysts over MgO, ZrO_2_, SiO_2_, TiO_2_ and Al_2_O_3_ were prepared to investigate the effect of the support materials on the physicochemical properties and catalytic performances for the conversion of MPC to effectively synthesize DPC. The catalysts were well characterized by XRD, BET, TEM, XPS, ICP-OES, H_2_-TPR, Py-IR and NH_3_-TPD. The results showed that the nature of the support obviously affected the structural properties and catalytic performances, and Pb was dispersed better on SiO_2_, TiO_2_, ZrO_2_ and MgO than on Al_2_O_3_, and showed stronger metal-support interaction over MgO and ZrO_2_. The activity results revealed that PbO/MgO and PbO/ZrO_2_ exhibited higher catalytic activities because they contained higher Pb dispersion and more Lewis acid sites, and the catalytic activities followed the order PbO/MgO > PbO/ZrO_2_ > PbO/SiO_2_ > PbO/Al_2_O_3_ > PbO/TiO_2_. On the contrary, PbO/MgO and PbO/ZrO_2_ exhibited better reusability due to strong interaction between the highly dispersed Pb and the supports, and the activity decrease in the case of PbO/SiO_2_, PbO/Al_2_O_3_ and PbO/TiO_2_ mainly resulted from the Pb leaching loss. This work would contribute to exploiting novel catalytic materials in a wide range of applications for the efficient synthesis of organic carbonates.

## Abstract 2

PbO/MgO and PbO/ZrO_2_ with a high number of Lewis acid sites act as efficient catalysts for MPC disproportionation.

## Graphical abstract

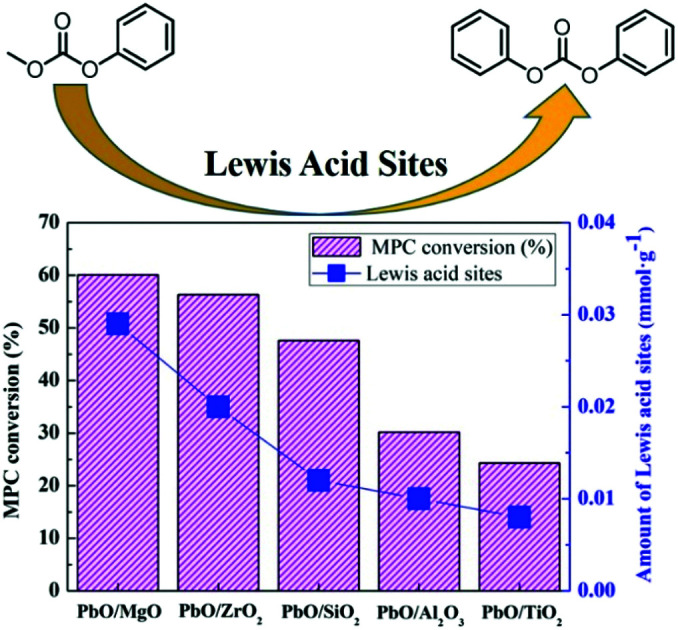

## 1. Introduction

**Scheme 1:**
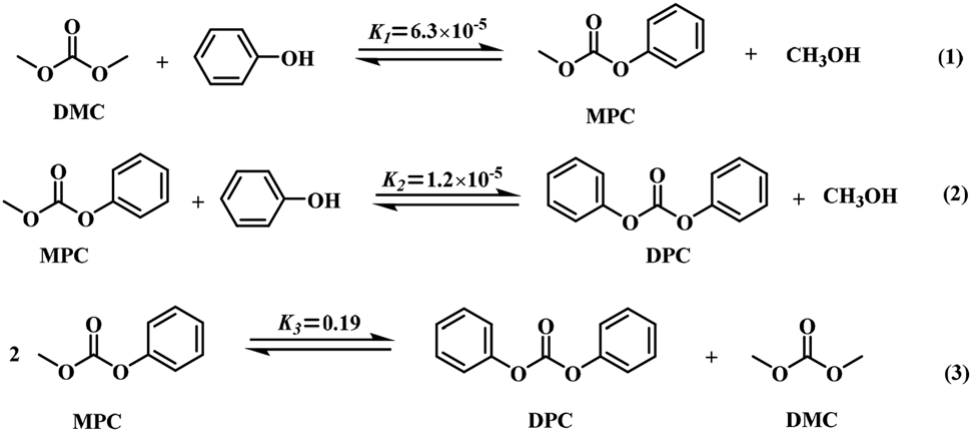
Transesterification process between DMC and phenol into DPC.

Due to its outstanding electrical, mechanical and heat resistance properties, polycarbonate (PC), as an important engineering thermoplastic for ubiquitous applications in several industries (automobiles, office equipment, medical devices, *etc.*), has attracted considerable interest in the last decades.^1,2^ Diphenyl carbonate (DPC), a building block, was often employed for the phosgene-free synthesis of PC by melt transesterification with bisphenol-A, and was also used for the production of pharmaceuticals, polymer materials, fine chemicals, *etc.*^3–5^ It was, therefore, necessary to develop efficient and environmentally benign synthetic routes to replace the highly toxic synthesis *via* phosgene. Among the alternative routes (phosgene method,^6,7^ oxidative carbonylation,^8,9^ transesterification,^10,11^*etc.*), the transesterification of phenol and dimethyl carbonate (DMC) was regarded as a most likely pathway to synthesize DPC without employing phosgene since the starting materials presented low toxicity and high biodegradability. This route consisted in two-step reaction where methyl phenyl carbonate (MPC) of the intermediate was first produced by the transesterification (Scheme 1-(1)), followed by the further transesterification reaction of MPC and phenol (Scheme 1-(2)) or the disproportionation of MPC (Scheme 1-(3)). Concerning the second-step reaction the equilibrium constants *K*_2_ and *K*_3_ indicated that DPC was mainly produced by the second step disproportionation instead of the further transesterification.^12,13^ On the contrary, the first step was rate-determining step since *K*_1_ was much lower than *K*_3_. Hence, considerable studies on the first step transesterification have been particularly carried out,^14–17^ but only few efforts have been made to the second step disproportionation.^18,19^ Indeed, the disproportionation of MPC is also the key step to produce DPC. Overall, it is very significant to intensively study on MPC disproportionation, which is advantageous to improving the yield of DPC and the conversion of the starting materials in future industrial applications.

The disproportionation reaction suffered from the low yield and selectivity of DPC due to the relatively low equilibrium constant and reaction rate. Therefore, the development of highly active catalysts is crucial in this reaction. Among various homogeneous catalytic systems reported like organo-tin/titanium catalysts, even though they exhibited outstanding catalytic activities,^19,20^ they had received little consideration because of the separation problems between DPC and the catalysts. Therefore, heterogeneous catalysis currently attracted more attention because these catalysts might be facilely recovered from the reaction mixture by simple filtration and could be recycled, making the procedure more feasible.

Recently, we studied the catalytic performances of various metal oxides including TiO_2_, MoO_3_, PbO and so on,^21–23^ and found that PbO was prepared into the supported Pb catalysts or mixed metal oxides significantly improved the catalytic activity, life time and stability.^24,25^ However, the nature and property of the support is often found to modify the distribution, loading, size, shape and electronic state of the active species as well as their interactions with support with some special physical–chemical properties, which is well known to strongly affect the catalytic activity and stability.^26–31^ Therefore, the study to investigate the structure–function relationship of the supports on Pb-based catalysts for MPC disproportionation is very worthwhile, which is conducive to screen suitable and promising supports.

In the present work, we formulate Pb-based catalysts by employing potential metal oxides as support phases and focus on studying the effect of various supports on the physicochemical properties of the catalysts and their catalytic performance to provide the role of support materials for developing efficient catalysis materials for MPC disproportionation to effectively synthesize DPC. Herein the Pb-based catalysts are prepared using wet-impregnation process over PbO loading onto SiO_2_, TiO_2_, Al_2_O_3_, MgO and ZrO_2_ supports. At the same time, the catalysts are thoroughly characterized by X-ray diffraction (XRD), temperature programmed reduction of H_2_ (H_2_-TPR), transmission electron microscopy (TEM), X-ray photoelectron spectrum (XPS), N_2_ adsorption/desorption measurement, pyridine-infrared spectroscopy (Py-IR) and temperature programmed desorption of NH_3_ (NH_3_-TPD) to clarify the support role on the Pb-based catalysts. In addition, the stabilities of these catalysts are also investigated in detail.

## 2. Experimental section

### 2.1 Material preparation

All chemicals adopted in this work were analytical purity, which were purchased from Sinopharm Chemical Reagent Co., Ltd and were directly used as received without further purification. Deionized water was used in all experimental synthesis procedures as needed.

Pure SiO_2_ was synthesized by sol–gel process. For this preparation, the desired amounts of tetraethyl orthosilicate, ethanol and distilled water were mixed together at room temperature. Then the ammonium aqueous solution was added gradually into the above solution under stirring at 50 °C to generate a transparent gel. The obtained gel was aged at 50 °C overnight, dried at 110 °C for 12 h and subsequently calcinated 550 °C for 5 h.

Pure TiO_2_ was prepared by hydrolysis process. Briefly, a proper amount of tetrabutyl titanate was dissolved in ethanol, and heated up to 50 °C under stirring. Then, appropriate amount of water was slowly added to promote the hydrolysis and was kept for 24 h. Finally, the sediment was filtered, washed with distilled water, and dried at 110 °C for 12 h and calcinated at 550 °C for 5 h.

Pure Al_2_O_3_ was prepared by a precipitation process. A certain amount of aluminum nitrate nonahydrate were dissolved in the deionized water and heated slowly to 90 °C. Afterwards, ammonia solution with 25% concentration was added into this solution under stirring until that the pH of 9–10 was reached. The as-prepared solid sediment was aged for 24 h and then vacuum filtered off and washed repeatedly. Finally, the obtained samples were dried at 110 °C for 12 h and subsequently calcinated at 550 °C for 5 h. Likewise, pure MgO and ZrO_2_ were prepared by using the similar process, respectively.

The preparation of supported Pb-based catalysts was carried out by incipient wetness impregnation process. Briefly, SiO_2_, TiO_2_, Al_2_O_3_, ZrO_2_ and MgO supports were homogeneously impregnated by using the aqueous solution of lead nitrate as precursor salt at ambient temperature for 24 h, respectively. After impregnation, the samples were dried in air at 110 °C for 12 h, and finally calcinated at 550 °C for 5 h. And the PbO loading used was 10 wt% for all the catalysts.

### 2.2 Material characterization

The X-ray powder diffraction (XRD) analysis was carried out on a DX-2700B diffractometer with the Ni-filtered CuKα radiation (1.5418 Å). The X-ray tube was operated at 40 kV and 30 mA, and the powder diffractogram was recorded at 0.02° intervals in the range of 5–90° with the scanning rate of 1.2° min^−1^.

The determination of the elemental composition was conducted by Optima 2000 DV inductively coupled plasma-optical emission spectrometer (PerkinElmer Inc., USA).

N_2_ adsorption–desorption isotherms were measured at liquid nitrogen temperature (−196 °C) using a 3H-2000PS2 adsorption instrument (BeiShiDe Instrument). Before the measurements, the catalysts were pretreated at 250 °C for 6 h under a high vacuum environment to remove physically adsorbed water. The specific surface area was calculated using the Brunauer–Emmett–Teller (BET) method and the pore volume and porous distribution was derived from Barrett–Joyner–Halenda (BJH) equation.

The morphology of the catalysts was investigated by transmission electron microscopy (JEM-1011) with a field-emission gun operating at 200 kV. The samples were prepared by dropping an ethanol suspension of the powder particles on a carbon film supported copper grid.

X-ray photoelectron spectrum (XPS) was employed to detect the chemical composition and states of the atoms on the catalysts, and conducted on a ESCALAB250Xi X-ray photoelectron spectrometer with C as the internal standard (C 1s = 284.7 eV).

H_2_-TPR experiment was carried out on a Micro TP-5076 chemisorption analyzer. Prior to the reduction, the catalyst (about 50 mg) was pretreated in N_2_ stream (20 mL min^−1^) at 300 °C for 1.5 h and then cooled to room temperature. After that, the flowing H_2_–N_2_ mixture (10% H_2_ by volume) was switched on, and the temperature was gradually increased from 50 to 800 °C with a ramp of 10 °C min^−1^. The H_2_ consumption was measured with a thermal conduction detector (TCD) that had been calibrated using the standard sample of CuO.

The number of acid sites was determined by NH_3_-TPD using the same instrument as that used for H_2_-TPR. Before the adsorption of NH_3_, the sample (about 100 mg) was first treated at 300 °C for 1.5 h and then decreased to 50 °C in a N_2_ flow of 20 mL min^−1^. After the saturated adsorption of NH_3_ (10 mL min^−1^) at 50 °C for 30 min, the physically adsorbed NH_3_ was subsequently degassed from the sample for 1 h under the N_2_ flow (20 mL min^−1^). Finally, the sample was heated to 800 °C with a rate of 10 °C min^−1^, the amounts of desorbed NH_3_ was monitored by the thermal conduction detector (TCD). The TPD profile was decomposed into Gaussian curves to quantify the weak, medium and strong acid sites.

Py-IR spectra were measured on a Thermo Nicolet 380 FT-IR spectrometer to determine the type of acid sites. Before the measurement, the impurity was removed from the sample under 10^−2^ Pa condition at 200 °C for 2 h, and then pyridine vapor was introduced into the sample for the saturated adsorption. After the evacuation at 200 °C for 1 h in flowing He atmosphere, the Py-IR spectra were subsequently collected at room temperature.

### 2.3 Catalytic reaction and the product analysis

MPC was synthesized by the reversible disproportionation of DMC and DPC, and its purity was 99.8% determined by high performance liquid chromatography.^20^ In a typical experiment, the catalytic performance test of MPC was performed in a three-necked glass beaker at atmospheric pressure. MPC of 150 mmol and a proper amount of the catalysts were placed into the beaker under a flowing nitrogen and slowly heated under vigorous stirring. When the reaction temperature was reached, it was kept for the reaction. As the reaction processed, DMC might be distilled out and continuously collected in a flask beaker to promote the equilibrium reaction towards the production of DPC. Upon the completion of the reaction, the reaction was decreased down to ambient temperature. The reaction mixture was analyzed by GC-MS instrument (HP-6890/5973) equipped with HP-5 capillary chromatography packed column. Subsequently, the products were quantitatively analyzed with a gas chromatograph (GC-7890A) equipped with HP-5 capillary column (30 m × 0.32 mm × 0.25 μm) and a flame ionization detector (FID).

## 3. Results and discussion

### 3.1 Characterization of the catalysts

#### 3.1.1 BET characterization

The BET surface area, pore volume and pore size distribution determined by nitrogen adsorption–desorption measurement for all the catalysts and their corresponding supports were listed in Table 1. The average pore diameters for these materials were in the mesoporous range, and the surface area and pore volume of the catalysts decreased in comparison to the corresponding supports. It was noted that the SiO_2_ support displayed the highest BET surface area among all support materials, moreover, after impregnating, the PbO/SiO_2_ catalyst also exhibited a larger BET specific surface area and pore volume in contrast to PbO/MgO, PbO/Al_2_O_3_, PbO/ZrO_2_ and PbO/TiO_2_, and the orders of both the surface area and pore volume for them were as follows: PbO/SiO_2_ > PbO/ZrO_2_ > PbO/MgO > PbO/TiO_2_ > PbO/Al_2_O_3_, in the same orders as that for the pure supports. The average pore diameters of the catalysts also decreased than those of the pure supports. This was probably due to PbO being dispersed on the surface of these supports or filled in the pore structure after the lead impregnating, which might result in the decrease of these textural properties.

**Table 1:** Results of BET measurements for the catalysts and corresponding supports

Catalysts	BET surface area (m^2^ g^−1^)	Total pore volume (cm^3^ g^−1^)	Average pore diameter (nm)
PbO/MgO	34.5	0.157	9.0
MgO	36.9	0.182	11.3
PbO/Al_2_O_3_	20.7	0.049	7.9
Al_2_O_3_	24.8	0.070	11.6
PbO/ZrO_2_	43.3	0.106	8.2
ZrO_2_	44.3	0.098	8.8
PbO/TiO_2_	30.3	0.073	9.9
TiO_2_	33.4	0.074	11.3
PbO/SiO_2_	67.3	0.522	11.1
SiO_2_	95.4	0.747	12.3

#### 3.1.2 XRD characterization

**Fig. 1:**
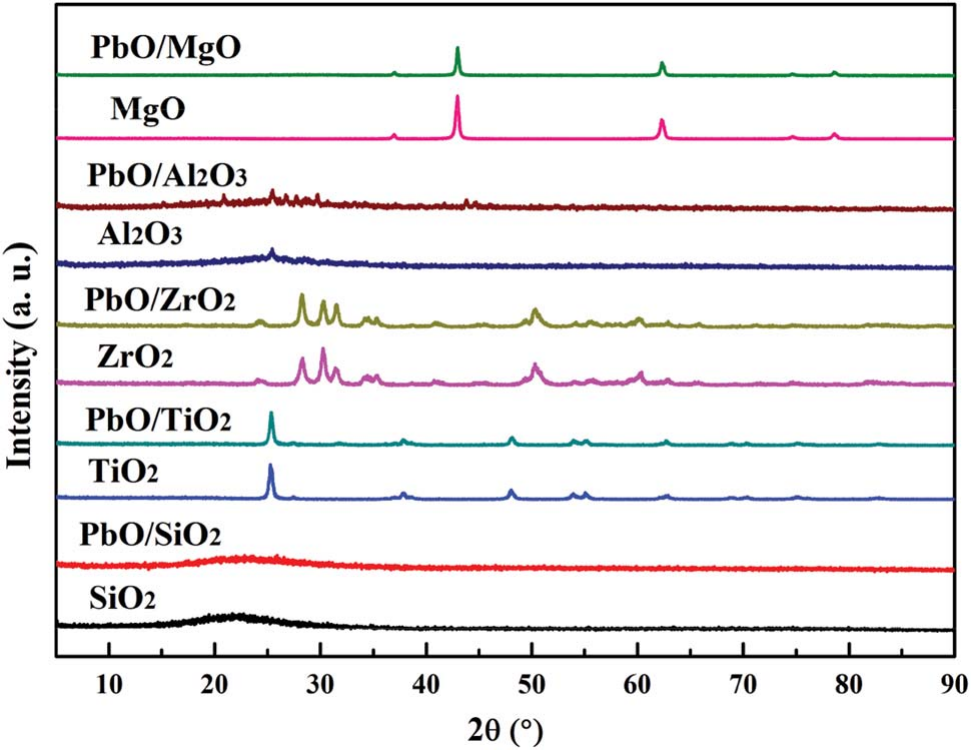
XRD patterns of the catalysts and corresponding supports.

To detect the structures of Pb species on various supports, XRD measurement were performed for both the catalysts and the pure supports. As could be seen in Fig. 1, XRD patterns of pure supports exhibited their respective characteristic peaks, while there were some differences with their corresponding Pb-based catalysts among the characteristic peaks. For instance, SiO_2_ exhibited a broad peak at around 22°, and after PbO loading on its surface no PbO crystalline phase diffraction peak was observed for PbO/SiO_2_. The diffraction peak at 25.4, 37.9, 48.1, 54.1 and 62.5° were attributed to anatase TiO_2_, and analogously the characteristic peak ascribed to PbO phases was absent for PbO/TiO_2_. ZrO_2_ displayed the existence of monoclinic (2*θ* = 30.3°) and tetragonal (2*θ* = 24.3 and 31.5°) zirconia phases, and PbO/ZrO_2_ exhibited the same structure with that of ZrO_2_ support. The diffraction peak at 25.5° belonging to γ-Al_2_O_3_ support was clearly observed,^32,33^ while PbO crystalline phases appeared at 20.8, 25.4, 26.6, 27.7 and 29.7° for PbO/Al_2_O_3_. PbO/MgO exhibited also the same crystalline structure with those of MgO support, and no PbO diffraction peak was observed. The XRD results suggested that the PbO active phases had better dispersion on the surface of SiO_2_, TiO_2_, ZrO_2_ and MgO supports in an amorphous state than that on Al_2_O_3_.

#### 3.1.3 H_2_-TPR characterization

**Fig. 2:**
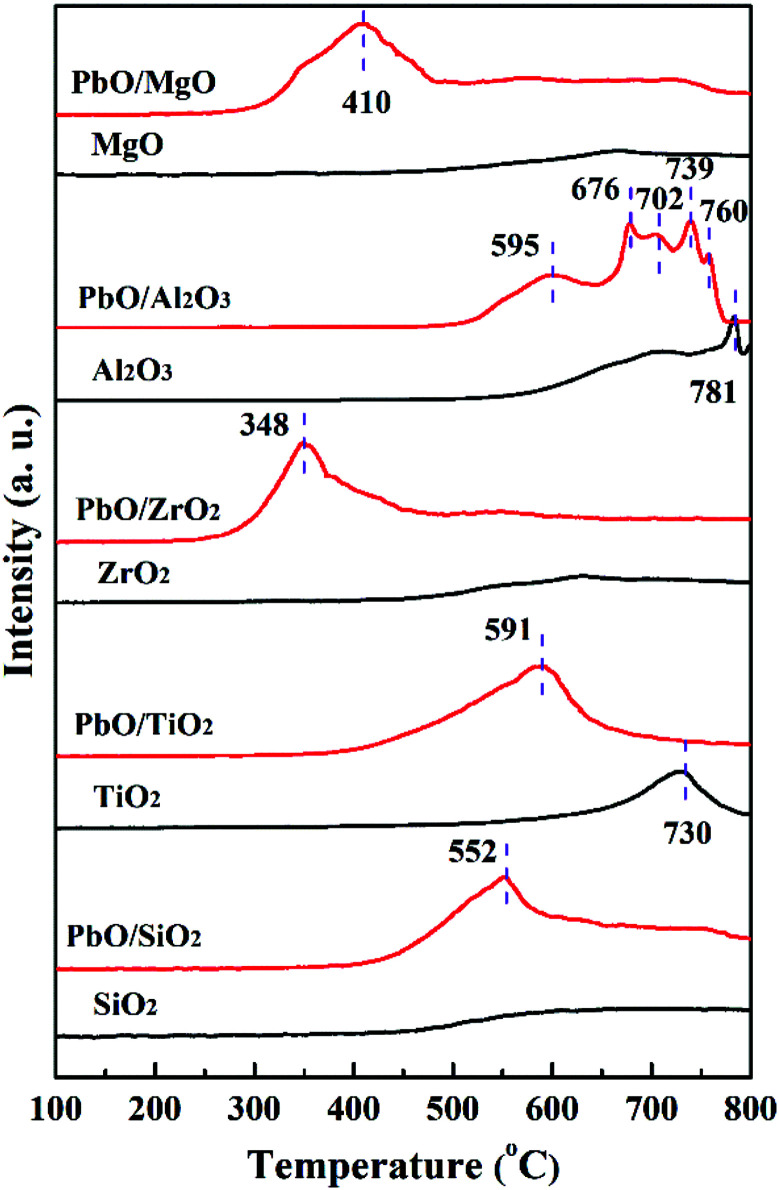
H_2_-TPR profiles of the catalysts and corresponding supports.

H_2_-TPR measurement was conducted to investigate the reduction behaviors of PbO species over the catalysts. The H_2_-TPR profiles of both the catalysts and pure supports were shown in Fig. 2. Firstly, for SiO_2_, ZrO_2_ and MgO supports, there were no evident reduction peak appearances in the temperature ranges of 100–800 °C. By contrast, the reduction characteristic peaks at 730 and 781 °C were observed for pure TiO_2_ and Al_2_O_3_, respectively. Secondly, for various Pb-based catalysts, only one distinct reduction characteristic peak was observed in PbO/SiO_2_, PbO/TiO_2_, PbO/ZrO_2_ and PbO/MgO respectively, suggesting that mainly PbO was reduced. As reported in the literatures,^25,34^ pure PbO revealed a clear reduction peak of 588 °C, which was assigned to PbO species reduction. Therefore, these peaks in PbO/SiO_2_ (552 °C), PbO/TiO_2_ (591 °C), PbO/ZrO_2_ (348 °C) and PbO/MgO (410 °C) were typically ascribed to the reduction of highly dispersed surface PbO species, nevertheless, it was noteworthy that there were also obvious differences in the peak positions for them, suggesting that PbO loading onto different supports had a great influence on the reduction temperature of PbO species. Notably, the reduction temperature of PbO species on SiO_2_, ZrO_2_ and MgO supports were shifted to lower temperatures. This was mainly due to the strong interaction between PbO species and the supports which was favorable for the stability of the catalysts during reaction. Whereas in the case of PbO/TiO_2_ the reduction peak shifted to a higher temperature and this was mainly due to the poor interaction between PbO and the TiO_2_ support. Thus, the results suggested that the interaction strengths between the active component and the different supports were remarkably different.

Thirdly, for PbO/Al_2_O_3_, five reduction characteristic peaks appeared. The first reduction peak at 595 °C corresponded to the surface PbO specie reduction, and the second reduction peak at 676 °C might result from the reduction of the bulk PbO, as the XRD results displayed the existence of PbO crystalline diffraction peaks, which was primarily assigned to the weak interaction between PbO and the Al_2_O_3_ support so that the PbO species was difficult to reduce at a higher reduction temperature. The following three peaks with reduction temperatures at 702, 739 and 760 °C were probably associated with the reduction of the Pb–Al mixed species formed *via* the interaction at high temperature (>700 °C) of partially reduced Pb-species and the support.^35,36^ Overall, according to the reduction temperatures of PbO species, it could be inferred that the interaction strengths between PbO and the supports were in the following order of PbO/ZrO_2_ > PbO/MgO > PbO/SiO_2_ > PbO/TiO_2_ > PbO/Al_2_O_3_, which might lead to different catalytic properties.

#### 3.1.4 TEM characterization

**Fig. 3:**
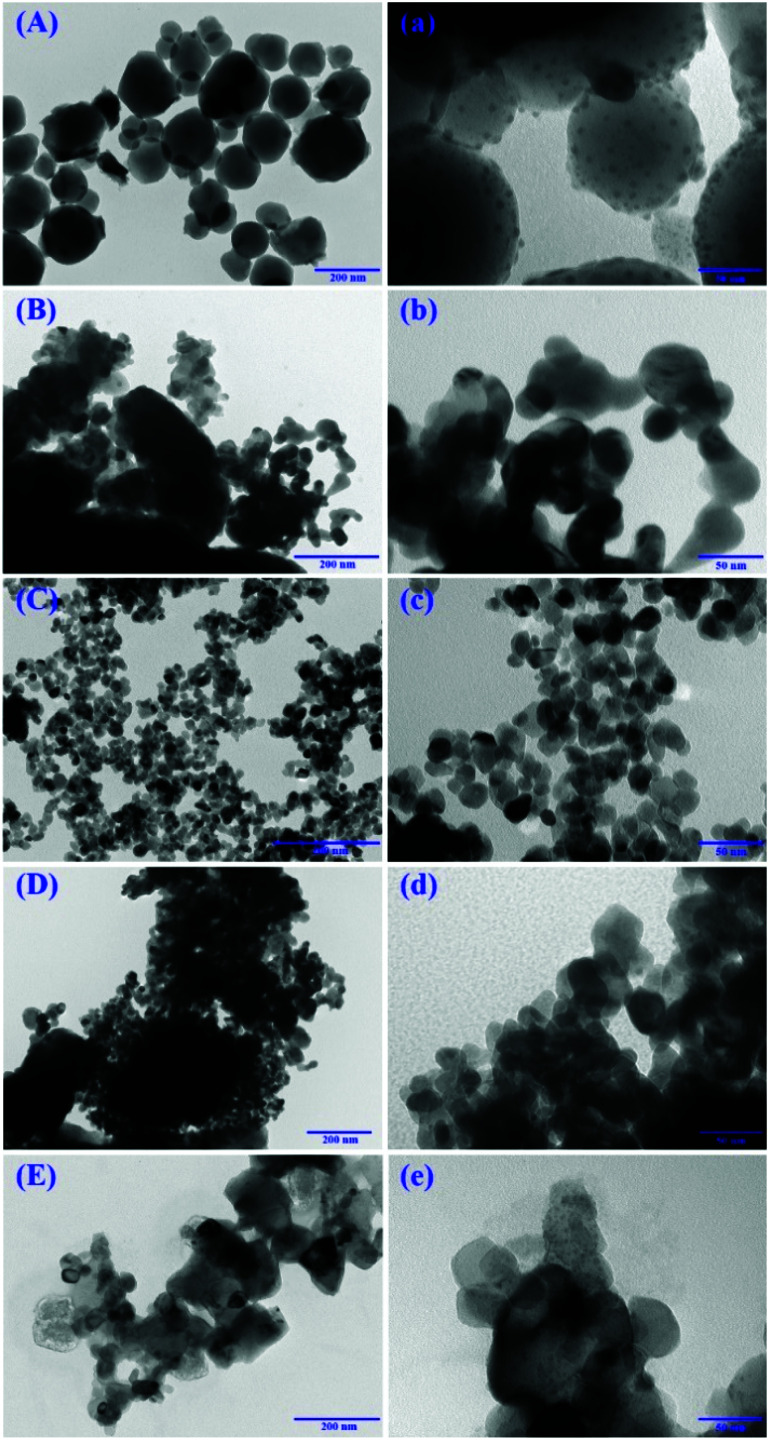
TEM pictures of PbO/SiO_2_ (A and a), PbO/TiO_2_ (B and b), PbO/ZrO_2_ (C and c), PbO/Al_2_O_3_ (D and d) and PbO/MgO (E and e).

Fig. 3 displayed the TEM images of the catalysts. As shown in Fig. 3(A and a), PbO/SiO_2_ mainly represented the spherical-like structure, and the PbO particles might be clearly observed, indicating that they were highly dispersed on the surface of the SiO_2_. For PbO/TiO_2_, PbO/ZrO_2_, PbO/Al_2_O_3_ and PbO/MgO displayed in Fig. 3(B, b)–(E, e), their morphology were remarkably different from each other. The diameters of all these catalysts were also not uniform, and the dissociation of PbO particles and the supports could not be detected by visual inspection. Comparatively speaking, PbO/ZrO_2_ exhibited a smaller particle size than the other catalysts.

#### 3.1.5 XPS characterization

**Fig. 4:**
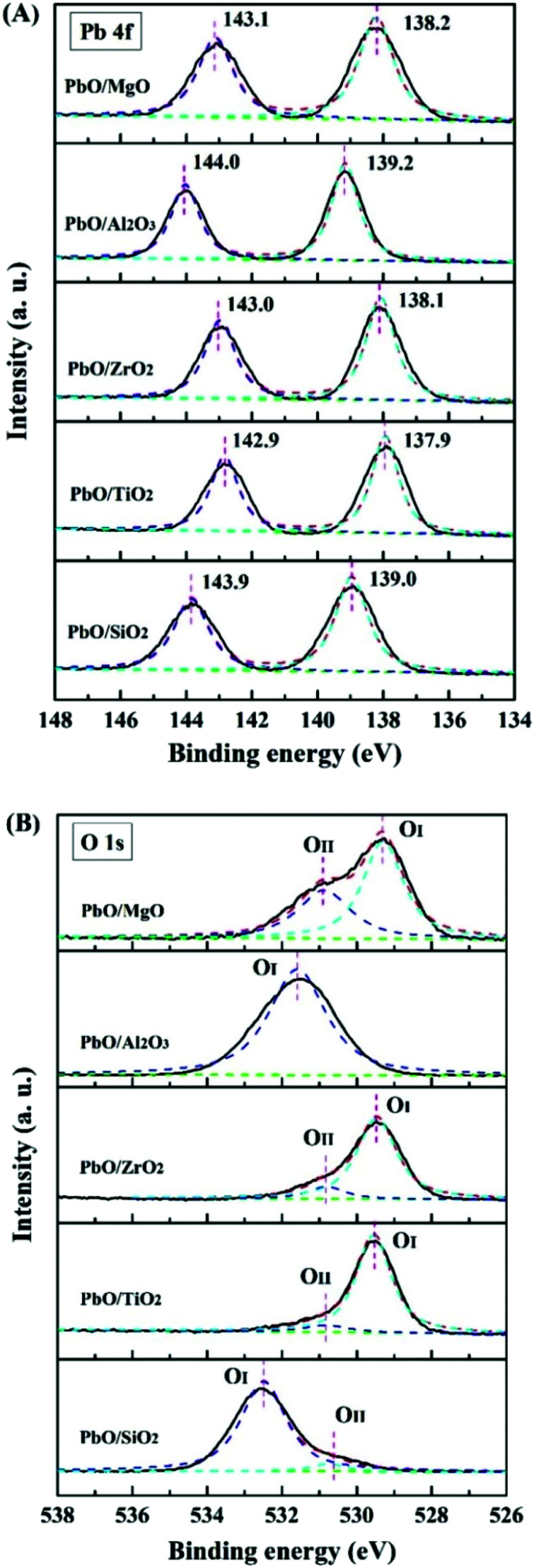
XPS spectra of the catalysts.

XPS analyses were employed to identify the chemical composition and states of the catalysts. The XPS survey spectrum verified the presence of Pb in the supported catalysts. As shown in Fig. 4(A), the definitive binding energy peak located at approximately 142.9–144.0 eV and 137.9–139.2 eV corresponded to Pb 4f_5/2_ and Pb 4f_7/2_,^23,37^ indicating that Pb^2+^ species existed on the surface of these catalysts. Fig. 4(B) showed that the O 1s spectra of the catalysts (except PbO/Al_2_O_3_ catalyst) could be deconvoluted into two Gaussian peaks (O_I_ and O_II_). In general, O_I_ was ascribed to the lattice oxygen in the literature,^28,34^ and O_II_ was related to the adsorbed oxygen species on the catalyst surface. The binding energy of the O 1s spectra was different in each catalyst, which was probably due to the different support properties for the supported Pb-based catalysts.

#### 3.1.6 NH_3_-TPD characterization

**Fig. 5:**
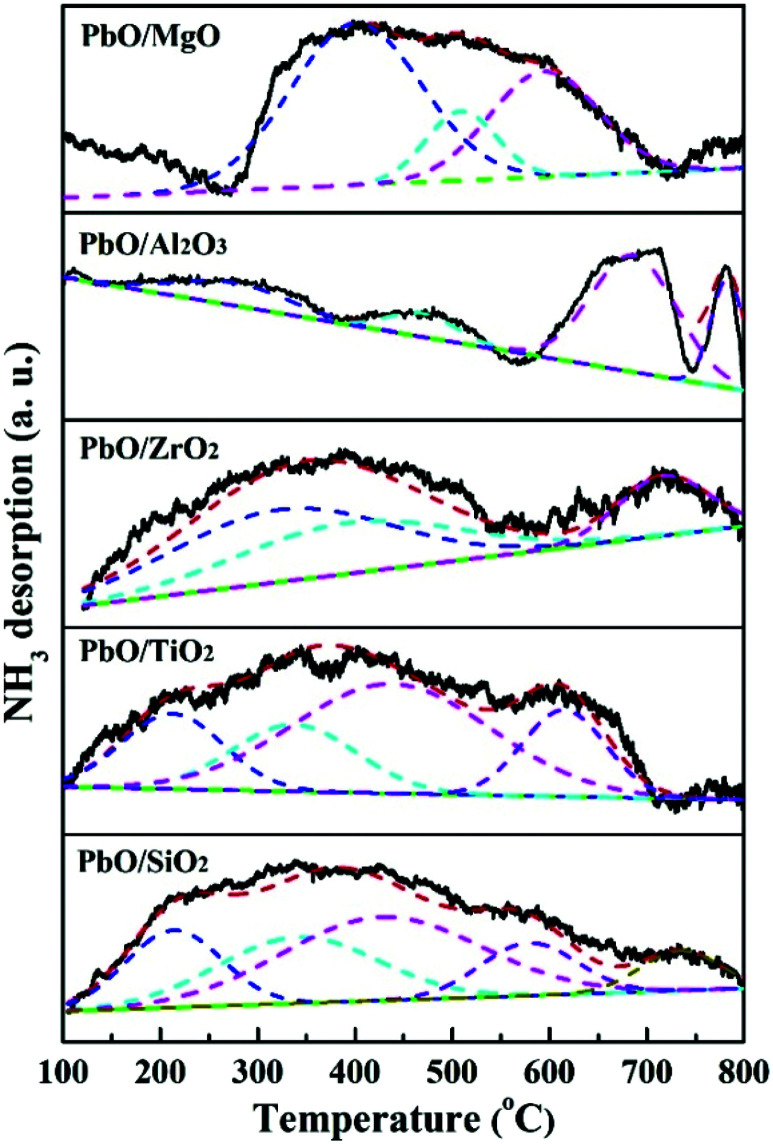
NH_3_-TPD profiles of the catalysts.

The process of MPC disproportionation to produce DPC was mainly dependent on the acidity of the catalyst. Herein NH_3_-TPD was carried out for measuring the acidic properties of the catalysts. The NH_3_-TPD profiles were displayed in Fig. 5. According to the literatures,^38,39^ the acid sites of the catalysts were usually classified based on desorption temperature range such as weak (around 100–200 °C), medium (around 200–400 °C), and strong (above 400 °C) acidic sites, respectively. As shown in Fig. 3, the acidity of the catalysts depended on the nature of the supports, and PbO/SiO_2_, PbO/TiO_2_ and PbO/ZrO_2_ exhibited remarkable and broad NH_3_ desorption peaks, suggesting the wide distribution of acid strengths from weak, medium and strong acid sites respectively. For PbO/MgO catalyst, only one strong high-temperature NH_3_ desorption peak at the range of 250 to 700 °C was detected, suggesting a strong acid property. PbO/Al_2_O_3_ showed much stronger NH_3_ desorption peak in the high-temperature region, indicating more strong acidic sites existed on the surface. Besides, the acidity data was also calculated and the results were given in Table 2. Clearly, the total acid amount in PbO/MgO was remarkably higher than those of other catalysts, and followed the sequence of PbO/MgO > PbO/ZrO_2_ > PbO/SiO_2_ > PbO/TiO_2_ > PbO/Al_2_O_3_. According to previous studies, the acidity of the catalysts was correlated with the activity, thus the high acidity of the catalyst was beneficial for the activity.

**Table 2:** The acidity of the catalysts determined by NH_3_-TPD

Catalysts	Acid amounts (mmol g^−1^)			Total acid amounts (mmol g^−1^)	*A*_L_/*A*_B_ ratio
	Weak	Medium	Strong		
PbO/MgO	0	0.030	0.048	0.078	0.582
PbO/Al_2_O_3_	0	0.001	0.009	0.010	—
PbO/ZrO_2_	0.004	0.019	0.029	0.052	0.610
PbO/TiO_2_	0.002	0.004	0.017	0.023	0.579
PbO/SiO_2_	0.002	0.014	0.020	0.036	0.489

#### 3.1.7 Py-IR characterization

**Fig. 6:**
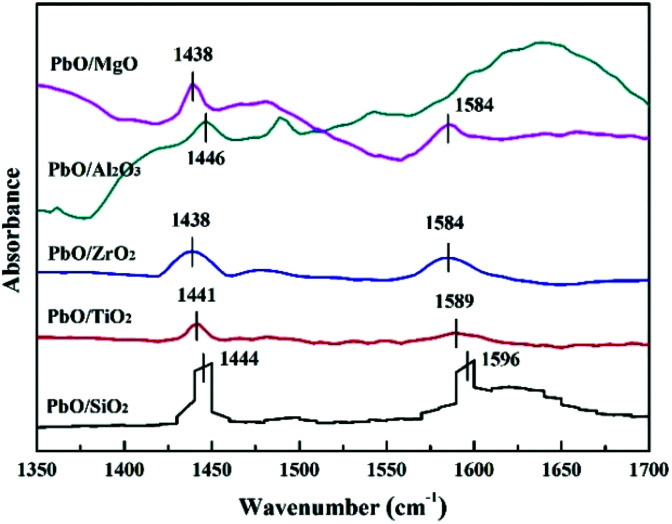
Py-IR spectra of the catalysts.

The type of acid sites was performed by pyridine adsorption FT-IR spectroscopy. Fig. 6 showed the Py-IR spectra of the catalysts. As shown in Fig. 6, PbO/SiO_2_, PbO/TiO_2_, PbO/ZrO_2_ and PbO/MgO exhibited two remarkable absorption peaks at around 1438–1446 and 1584–1596 cm^−1^, which were attributed to Lewis and Brønsted acidic sites, respectively.^40,41^ However, for PbO/Al_2_O_3_, only one absorption peak was displayed at 1446 cm^−1^ that suggested the presence of only Lewis acidic sites. Meanwhile, the concentration ratio of Lewis to Brønsted acid sites was calculated by semiquantitative analysis based on the relative peak area corresponding to Lewis and Brønsted acidity (*A*_L_/*A*_B_) in Table 2. Apparently, the *A*_L_/*A*_B_ ratios were higher for PbO/ZrO_2_ and PbO/MgO compared to others, and combined with the acid amount results determined by NH_3_-TPD, which indicated that PbO/ZrO_2_ and PbO/MgO contained much more Lewis acidic sites.

### 3.2 Results of the catalytic performance evaluation

**Fig. 7:**
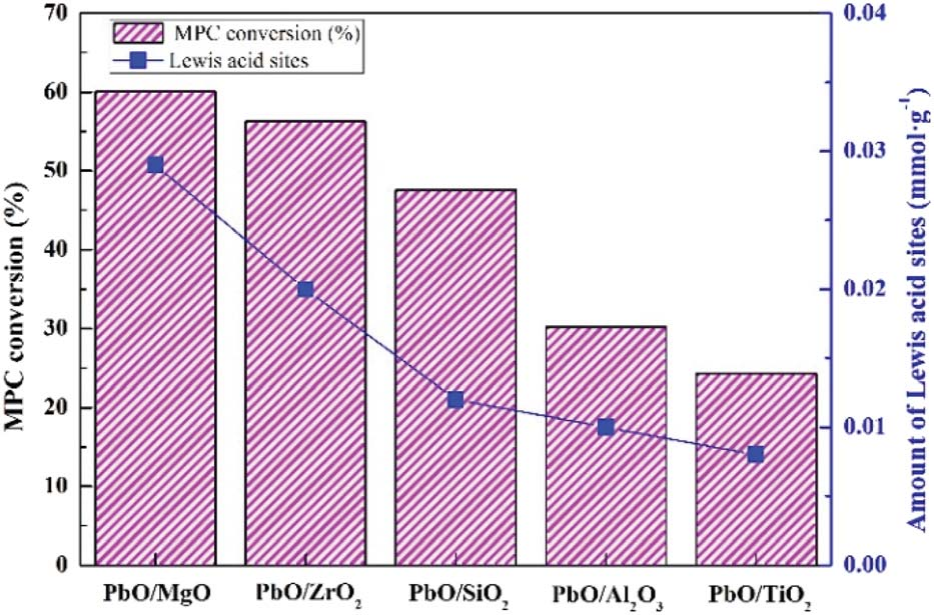
MPC conversion and Lewis acid sites on the catalysts.

The catalytic performance evaluation results for MPC disproportionation over the catalysts were revealed in Table 3. Pure supports were basically inactive under the current studies, while PbO loading on these supports exhibited considerably different catalytic performances. The catalytic activities of PbO/MgO and PbO/ZrO_2_ were close and obviously higher than those of PbO/SiO_2_, PbO/Al_2_O_3_ and PbO/TiO_2_, and the sequence of the activities for the MPC conversion was as follows: PbO/MgO > PbO/ZrO_2_ > PbO/SiO_2_ > PbO/Al_2_O_3_ > PbO/TiO_2_. Previous studies displayed that the BET surface area and acidity of catalyst played significant role in the catalytic activity and product selectivity. Nevertheless, this order of the activity was not correlated with the sequence of BET surface area, indicating that the surface area might be not a main factor determining the activity. Therefore, the catalytic activity might be closely related to the acidity. Combining with the characteristic results of Py-IR and NH_3_-TPD, the relationship between the activity and Lewis acidity was displayed in Fig. 7. It was clear that the activity depended on the acidic site, and the more Lewis acid site was, the higher the activity was. And the decrease sequence of MPC conversion was in accordance with the order of the Lewis acid site, suggesting that the Lewis acid was the critical factor to determine the catalytic activity. What's more, although the highest DPC yield was obtained over PbO/MgO due to the acidity of this catalyst being the highest among all the catalysts, yet it was also noteworthy that a strong acid strength could induce the decarboxylation decomposition of MPC into the byproduct anisole. Therefore, it could be ascertained that the higher anisole content was mainly due to its excessively strong acidity.^20^ Moreover, PbO/Al_2_O_3_ with more strong acid strength exhibited the higher anisole content, which further testified the reason. Thus, the results also suggested that medium and moderately strong acidic sites were favorable for the higher DPC selectivity.

**Table 3:** Catalytic performance results of MPC disproportionation over the catalysts^a^

Catalysts	MPC conversion/%	Yield/%		DPC selectivity/%
		DPC	Anisole	
PbO/MgO	60.1	56.0	4.1	93.2
PbO/ZrO_2_	56.3	55.2	1.1	98.0
PbO/SiO_2_	47.7	46.2	1.6	96.9
PbO/Al_2_O_3_	30.2	26.5	3.7	87.7
PbO/TiO_2_	24.3	22.9	1.4	94.2

a Reaction conditions: MPC (150 mmol), catalyst (1.2 g), 200 °C, 2.5 h. Anisole was a small amount of byproduct.

### 3.3 Recyclability of the catalysts

**Fig. 8:**
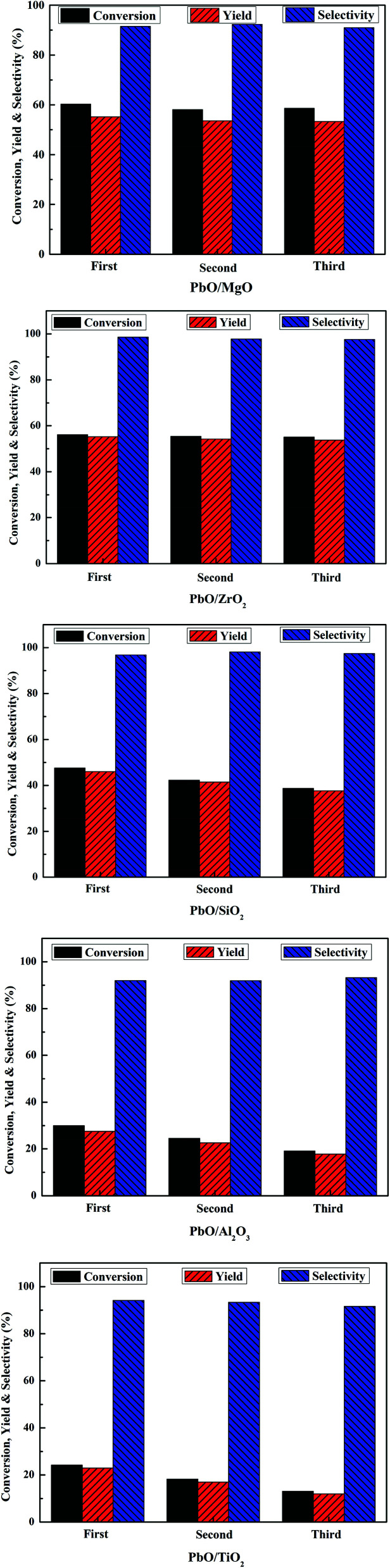
Reusability of the catalysts. Reaction conditions: MPC (150 mmol), catalyst (1.2 g), 200 °C, 2.5 h.

Recycling experiments of the catalysts were performed to check the stability. After each run, the catalysts were separated by filtration, washed thoroughly with DMC and dried in vacuum at 110 °C overnight, and then reused for next cycle reaction. As displayed in Fig. 8, there was no remarkable change in the MPC conversion and yield DPC after three cycles over PbO/MgO and PbO/ZrO_2_. However, for PbO/SiO_2_, PbO/Al_2_O_3_ and PbO/TiO_2_ catalysts, the catalytic activities decreased gradually in the succeeding three runs, suggesting that PbO/MgO and PbO/ZrO_2_ possessed much better reusability than PbO/SiO_2_, PbO/Al_2_O_3_ and PbO/TiO_2_.

In heterogeneous catalysis, the leaching of active component was often the main reason which decreased the activity for further reuse. Therefore, the recovered catalysts were detected by ICP-OES analysis (Table S1†), and the content of Pb in the fresh and the third reused catalyst showed that Pb content had no significant loss over PbO/MgO and PbO/ZrO_2_, but dropped markedly over PbO/SiO_2_, PbO/Al_2_O_3_ and PbO/TiO_2_, respectively. Hence, the leaching loss of Pb might lead to the decrease of catalytic activities for the recovered PbO/SiO_2_, PbO/Al_2_O_3_ and PbO/TiO_2_ catalysts. Combined with the characteristic results of H_2_-TPR, the excellent recyclability of PbO/MgO and PbO/ZrO_2_ was possibly attributed to their strong interactions of the supports with highly dispersed Pb, which was potentially important for practical applications. Besides, the recovered catalysts after the third use were also detected by XRD characterization (Fig. S1†). The result indicated that the diffraction peaks of the third reused catalysts were basically identical to those of the fresh catalysts, suggesting that with the exception of PbO/Al_2_O_3_, PbO was still amorphous or microcrystalline states in the recovered catalysts. Therefore, MgO and ZrO_2_ might be suitable and promising support materials for the better preparation of Pb-based catalysts to transform MPC disproportionation to efficiently synthesize DPC in comparison with SiO_2_, Al_2_O_3_ and TiO_2_.

## 4. Conclusions

In summary, the support nature modified the structural properties of Pb catalysts and exerted marked influence on their catalytic performances. The catalysts were characterized by physicochemical techniques, and the results showed that PbO was dispersed better on SiO_2_, TiO_2_, ZrO_2_ and MgO than Al_2_O_3_, and the stronger metal-support interaction was obtained over MgO and ZrO_2_. The active results showed that the catalytic performances over MgO and ZrO_2_ to load Pb catalysts were significantly superior to those of SiO_2_, Al_2_O_3_ and TiO_2_, because of the higher active component dispersion and more Lewis acid active sites. Furthermore, the excellent recyclability in the case of PbO/MgO and PbO/ZrO_2_ was potentially important in industrial application. Thus, from this study it could be noted that the more Lewis acid site was present, the more the activity was, and the nature of the support was a key parameter for MPC disproportionation.

## Conflicts of interest

There are no conflicts to declare.

## Supplementary Material

RA-009-C9RA03931G-s001
