# Supplementary material for: Highly effective transformation of methyl phenyl carbonate to diphenyl carbonate with recyclable Pb nanocatalyst
Source: RSC Adv. 2019 Jul 1;9(35):20415–23. doi: 10.1039/c9ra03931g (PMC9065496; doi:10.1039/c9ra03931g)
Supplement: RA-009-C9RA03931G-s001 [file RA-009-C9RA03931G-s001.pdf]

Electronic Supplementary Material (ESI) for RSC Advances.
This journal is © The Royal Society of Chemistry 2019

Highly effective transformation of methyl phenyl carbonate to diphenyl carbonate with recyclable Pb nanocatalyst

Songlin Wang [a, b], Hongying Niu [a], Jianji Wang [b], Tong Chen [c, *], Gongying Wang [c], Jiamin Zhang [a]

[a] School of Chemistry and Chemical Engineering, Henan Institute of Science and Technology, Xinxiang 453003, China

[b] Postdoctoral Programs, Key Laboratory of Green Chemical Media and Reactions, Ministry of Education, School of Chemistry and Chemical Engineering, Henan Normal University, Xinxiang 453007, China

[c] Chengdu Institute of Organic Chemistry, Chinese Academy of Sciences, Chengdu 610041, China

Corresponding author. E-mail: chentongw@sina.com.cn (T. Chen)

Table S1 The content of Pb in the fresh and used catalysts.

Catalyst	PbO content (wt. %)	
	Fresh	Used[a]
PbO/MgO	9.86	9.61
PbO/ZrO_2	9.49	9.40
PbO/SiO_2	9.93	8.84
PbO/Al_2O_3	9.88	8.65
PbO/TiO_2	9.63	8.40

[a] After the third reused catalyst.

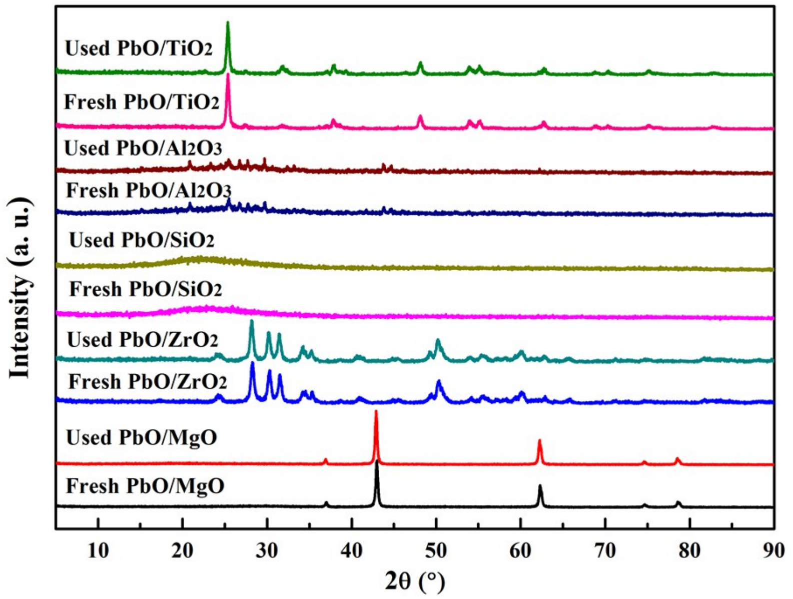

Fig. S1. XRD patterns of fresh and recovered catalysts after the third use.
